# Comparative Retrospective Analysis of Cross-Finger Flap Outcomes: A Study on Split-Thickness Skin Graft vs. Full-Thickness Skin Graft in Donor Fingers

**DOI:** 10.7759/cureus.72013

**Published:** 2024-10-21

**Authors:** Arun Prasath S., Santharam R., Balasubramanian C.

**Affiliations:** 1 Plastic Surgery, SRM Medical College Hospital and Research Center, Chennai, IND

**Keywords:** cross-finger flap, donor site morbidity, finger injuries, retrospective study, skin graft

## Abstract

Introduction: Hand injuries, particularly those involving the fingers, are complex and often necessitate meticulous surgical interventions. Cross-finger flaps (CFFs) are a reliable technique for covering finger defects, with the choice of skin graft at the donor site playing a crucial role in the procedure's success. Split-thickness skin grafts (STSGs) and full-thickness skin grafts (FTSGs) are commonly used, each offering distinct advantages and drawbacks. This study compares the functional and aesthetic outcomes of donor fingers covered with STSG versus FTSG in CFF procedures.

Materials and methods: This retrospective observational study was conducted from January 2020 to 2024. A total of 82 patients who underwent CFF surgery were included, with 41 patients each in Group A (STSG) and Group B (FTSG). The functional and aesthetic outcomes were assessed. Statistical analysis was performed using SPSS version 26.0 (IBM Corp., Armonk, NY), with significance set at p < 0.05.

Results: The study revealed that Group B had superior outcomes across all measured parameters. The mean visual analog scale (VAS) score for aesthetic outcomes was significantly higher in Group B (8.5 ± 1.2) compared to Group A (7.0 ± 1.5, p < 0.01). Functional recovery in proximal interphalangeal (PIP) and distal interphalangeal (DIP) joints, measured by range of motion, was also better in Group B compared to Group A (p = 0.002). Sensory recovery was more favorable in Group B, with 85% achieving S3+ or better, compared to 60% in Group A (p < 0.05). Additionally, the graft donor site scar was significantly less noticeable in Group B, with a Vancouver Scar Scale score of 3.5 ± 1.1, compared to 5.0 ± 1.4 in Group A (p < 0.01).

Conclusion: FTSG offers superior functional and aesthetic outcomes compared to STSG in this study. The findings suggest that FTSG should be preferred for covering donor fingers. These results provide strong evidence for the use of FTSG in optimizing surgical outcomes and improving patient satisfaction.

## Introduction

Hand injuries, particularly those involving the fingers, are common and often result in complex wounds requiring meticulous surgical intervention. Cross-finger flaps (CFFs) have become a reliable method for covering defects in the fingers, particularly when the primary goal is to preserve function and aesthetics. The choice of the donor site and the method of wound closure at the donor site are critical factors that influence the overall success of the procedure. Traditionally, donor site defects are covered using either split-thickness skin grafts (STSGs) or full-thickness skin grafts (FTSGs). Each technique has its advantages and drawbacks, which can significantly affect the outcomes related to healing, aesthetics, and long-term functionality [1].

The choice between STSG and FTSG is influenced by various factors, including the size and location of the donor site, the patient's condition, and the surgeon's preference. STSG is often favored for its ease of harvest and greater availability of donor skin, making it suitable for larger defects [2].

However, STSG may be associated with complications such as more significant donor site morbidity, including pain, scarring, and potential wound healing issues due to the thinner graft. In contrast, FTSG, though limited by the availability of donor skin and technically more demanding, is often associated with better aesthetic outcomes and reduced contracture, particularly in areas of high mobility such as the fingers.

CFF techniques were first introduced in the 1950s as a method to address complex fingertip and volar defects, which are challenging to manage due to the intricate anatomy and functional importance of the digits. Over time, these techniques have been refined, and their indications expanded. Donor site morbidity is a significant concern in CFF procedures, as poor healing or significant scarring can impair hand function and cause considerable patient discomfort. Thus, the choice of skin graft for the donor site has been a subject of considerable debate in hand surgery literature [3].

Studies comparing STSG and FTSG in various anatomical sites have shown differing outcomes regarding graft survival, aesthetic appearance, and functional recovery. However, there is limited high-quality evidence focusing specifically on donor sites in CFF procedures. Understanding the comparative outcomes between STSG and FTSG in this context is crucial for optimizing surgical techniques and improving patient outcomes [4].

This study aims to conduct a comparative retrospective analysis of CFF outcomes with a specific focus on the differences between STSGs and FTSGs used for covering the donor site. By retrospectively analyzing patient records and outcomes, this study seeks to provide evidence-based insights that could guide the choice of grafting technique in CFF procedures.

The rationale for this study is grounded in the need to optimize functional and aesthetic outcomes for patients undergoing CFF procedures. Given the importance of hand function in daily life, minimizing donor site morbidity while ensuring the durability of the flap is essential. This study hypothesizes that FTSG may offer superior outcomes in terms of reduced donor site morbidity and better overall patient satisfaction compared to STSG. The results of this analysis could potentially lead to a shift in clinical practice, favoring one technique over the other based on objective outcome measures.

## Materials and methods

This study was a retrospective observational analysis conducted between January 2020 and January 2024 in the Department of Plastic Surgery at SRM Medical College and Research Center, Kattankulathur. This study included 82 patients who had undergone CFF surgery and had completed the follow-up period of one year following flap division. The study population was divided into two groups: Group A - where the donor finger defect was covered with a STSG and Group B - where an FTSG was used.

Inclusion criteria were set to include only those patients who had completed the follow-up, while patients with incomplete follow-up or medical records were excluded. Data was extracted from inpatient and outpatient records using a standardized form. The clinical assessment included the type and extent of finger injury, radiographic evaluation, and pre-operative clinical photography with consent.

The surgical procedures varied between the two groups, with STSG harvested from the ipsilateral arm or thigh for Group A and FTSG harvested from the lateral inguinal crease or ipsilateral inguinal crease for Group B. Post-operatively, patients were discharged on the second day with a plaster of Paris (POP) dressing, with flap division on the 14th day. The follow-up protocol was as follows: Suture removal on the 10th post-operative day following flap division. The rehabilitation program included Motor (Active and Passive range of motion), sensory rehabilitation (sensory assessment and reeducation), and scar management (scar massage with silicone gel). Initially following suture removal rehabilitation was done daily for the first two weeks and once weekly (along with a home exercise program) for the next six weeks. After the first eight weeks, monthly follow-up from the third month to the 12th month - during which sensory rehabilitation and scar management were continued. The tools used for assessment in the study included the following:

Goniometer

This tool (Ramé-hart, NJ) was utilized to measure the range of motion in the affected and donor fingers.

Power assessment

FDP/FDS (MRC Grading)

The strength of the flexor digitorum profundus (FDP) and flexor digitorum superficialis (FDS) muscles was evaluated using the Medical Research Council (MRC) grading system.

Sensory recovery

Semmes-Weinstein Monofilament Test

This test was used to assess the sensory recovery of the donor finger by evaluating the touch-pressure threshold.

Two-Point Discrimination Score

This score was employed to measure the ability of the patient to distinguish between two closely spaced points on the skin, indicating sensory recovery.

Aesthetic outcome

Visual Analog Scale (VAS)

This scale was used to assess the aesthetic outcome of the donor finger from the patient’s perspective.

Vancouver Scar Scale

This scale was utilized to evaluate the scar quality at the graft donor site, considering factors such as pigmentation, vascularity, pliability, and height.

The statistical analysis was done using SPSS version 26.0 (IBM Corp., Armonk, NY). Continuous variables, such as range of motion, graft survival rate, and scar assessment scores, were reported as mean ± standard deviation and analyzed using independent samples t-tests. Categorical variables, including graft complications and overall satisfaction, were analyzed using Chi-square tests. For non-normally distributed continuous data, the Mann-Whitney U test was applied. Repeated measures ANOVA was used to evaluate changes in functional recovery, power, and sensory recovery over time within each group. Additionally, logistic regression analysis was conducted to identify predictors of graft complications and poor aesthetic outcomes, with adjustments made for covariates such as age, gender, and graft donor site. A p-value of less than 0.05 was considered statistically significant.

## Results

The total sample size was 82, with 41 patients in each group (Group A: STSG, Group B: FTSG). The baseline demographic characteristics (Table 1) of the two groups, Group A (STSG) and Group B (FTSG), were well balanced, with no significant differences between the groups. The mean age of patients in Group A was 34.5 ± 8.3 years, while in Group B, it was 35.2 ± 9.1 years. The gender distribution was similar, with 71% males and 29% females in Group A, compared to 73% males and 27% females in Group B. Both groups had a comparable occupational distribution, with the majority being laborers, followed by carpenters, teachers, students, and others. Right-hand dominance was prevalent in both groups, with 85% in Group A and 83% in Group B. The injury sites were also similarly distributed across both groups, with the index finger being the most commonly affected, followed by the middle, ring, and little fingers.

**Table 1:** Baseline demographic characteristics

Demographic variable	Group A (STSG), n=41	Group B (FTSG), n=41
Age (years) (Mean ± SD)	34.5 ± 8.3	35.2 ± 9.1
Gender		
Male (n%)	29 (71)	30 (73)
Female (n%)	12 (29)	11 (27)
Occupation		
Laborers (n%)	18 (44)	19 (46)
Teachers (n%)	6 (15)	5 (12)
Students (n%)	5 (12)	4 (10)
Carpenters (n%)	7 (17)	8 (20)
Others (n%)	5 (12)	5 (12)
Dominance		
Right-hand dominant (n%)	35 (85)	34 (83)
Left-hand dominant (n%)	6 (15)	7 (17)
Injury site		
Index finger (n%)	16 (39)	15 (37)
Middle finger (n%)	14 (34)	13 (32)
Ring finger (n%)	8 (20)	9 (22)
Little finger (n%)	3 (7)	4 (10)

**Figure 1:**
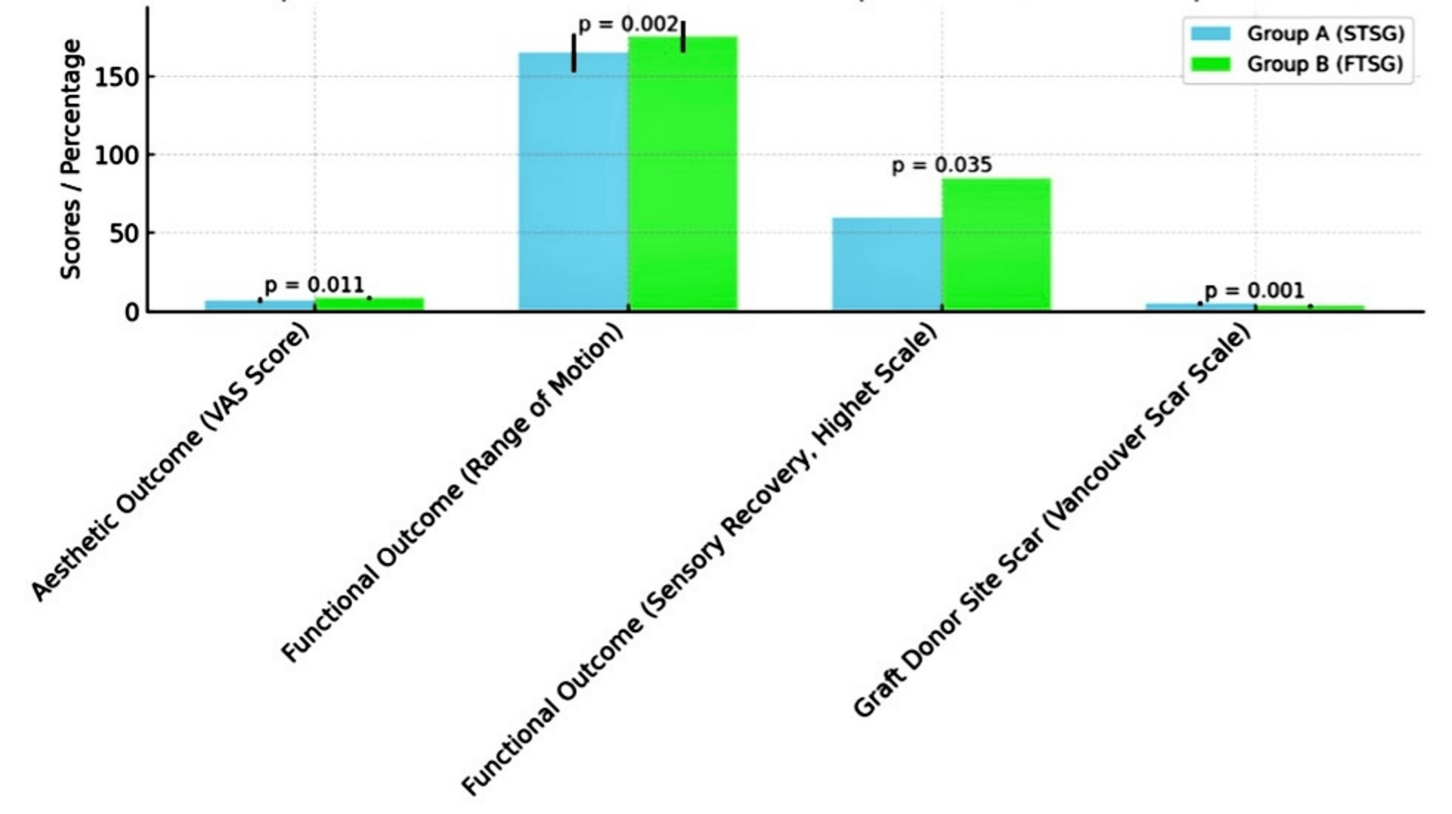
Comparison of outcomes between Group A (STSG) and Group B (FTSG) STSG - Split Thickness Skin Graft, FTSG - Full Thickness Skin Graft, VAS - Visual Analog Scale The statistical tests used were t-tests for continuous variables (mean ± SD) and chi-square test for categorical proportions (e.g., Sensory Recovery) Chi-square test value for sensory recovery (categorical proportions): 4.39 T-tests value for continuous variables (asthetic outcome: -5.74, proximal and distal interphalangeal joint range motion:  -9.13, -4.00, graft donor site scar: 3.53) P-value of less than 0.05 was considered statistically significant.

The comparison of aesthetic and functional outcomes (Table 2, Figure 1) between the two groups, showed that Group B (FTSG) had superior outcomes in all measured parameters. The aesthetic outcome, as assessed by the VAS score, was significantly better in Group B, with a mean score of 8.5 ± 1.2 compared to 7.0 ±1.5 in Group A (p < 0.01). Functional recovery, measured by the range of motion, was also better in Group B compared to Group A, with a p-value of 0.002. Sensory recovery, evaluated using the Highet scale, showed that 85% of patients in Group B achieved S3+ or better, compared to 60% in Group A (p < 0.05). Additionally, the graft donor site scar was significantly less noticeable in Group B, with a Vancouver Scar Scale score of 3.5 ± 1.1, compared to 5.0 ± 1.4 in Group A (p < 0.01).

**Table 2:** Comparison of aesthetic and functional outcomes between the two groups VAS - Visual Analog Scale, PIP - Proximal Interphalangeal Joint, DIP - Distal Interphalangeal Joint The statistical tests used were t-tests for continuous variables (mean ± SD) and chi-square test for categorical proportions (e.g., Sensory Recovery). P-value of less than 0.05 was considered statistically significant.

Outcome	Group A (STSG) (Mean ± SD)	Group B (FTSG) (Mean ± SD)	Test statistic	P-value
Aesthetic outcome (VAS Score)	7.0 ± 1.5	8.5 ± 1.2	-5.74	0.011
Functional outcome				
PIP (range of motion)	85.3° ± 10.4°	105.5° ± 8.2°	-9.13	0.002
DIP (range of motion)	70.5° ± 12.2°	85.4° ± 10.6°	-4.00	0.001
Functional outcome (sensory recovery, Highet Scale)	60% S3+ or better	85% S3+ or better	4.39	0.035
Graft donor site scar (Vancouver Scar Scale)	5.0 ± 1.4	3.5 ± 1.1	3.53	0.001

**Figure 2:**
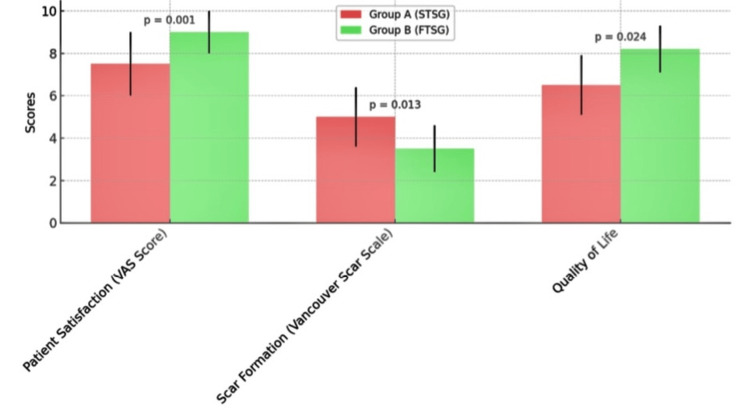
Comparison of patient-related outcomes between Group A (STSG) and Group B (FTSG) STSG - Split Thickness Skin Graft, FTSG - Full Thickness Skin Graft, VAS - Visual Analog Scale t-tests were used to compare the means of patient-related outcomes between the two groups (Group A and Group B) P-value of less than 0.05 was considered statistically significant.

Patient-related outcomes (Table 3, Figure 2) further reinforced the superiority of Group B (FTSG) over Group A (STSG). Patient satisfaction, as measured by the VAS score, was significantly higher in Group B, with a mean score of 9.0 ± 1.0 compared to 7.5 ± 1.5 in Group A (p < 0.01). Scar formation, assessed using the Vancouver Scar Scale, also favored Group B, with a lower mean score of 3.5 ± 1.1 compared to 5.0 ± 1.4 in Group A (p < 0.01). Quality of life was reported to be higher in Group B, with a mean score of 8.2 ± 1.1, while Group A had a mean score of 6.5 ± 1.4 (p < 0.01), indicating a significant positive impact of FTSG on patient well-being.

**Table 3:** Comparison of patient-related outcomes between the two groups STSG - Split Thickness Skin Graft, FTSG - Full Thickness Skin Graft, VAS - Visual Analog Scale t-tests were used to compare the means of patient-related outcomes between the two groups (Group A and Group B) P-value of less than 0.05 was considered statistically significant.

Outcome	Group A (STSG) (Mean ± SD)	Group B (FTSG) (Mean ± SD)	p-value
Patient Satisfaction (VAS Score)	7.5 ± 1.5	9.0 ± 1.0	0.001
Scar Formation (Vancouver Scar Scale)	5.0 ± 1.4	3.5 ± 1.1	0.013
Quality of Life	6.5 ± 1.4	8.2 ± 1.1	0.024

The comparison of complications (Table 4) revealed that Group B (FTSG) experienced fewer graft-related complications compared to Group A (STSG). The rate of uptake failure was lower in Group B (5%) than in Group A (15%), with a statistically significant difference (p < 0.05). Similarly, the incidence of infection was 3% in Group B and 5% in Group A (p < 0.05). Contracture formation was also less common in Group B, occurring in 2% of patients compared to 5% in Group A (p < 0.05). These results indicate that FTSG is associated with a lower risk of complications compared to STSG.

**Table 4:** Comparison of complications between the two groups Chi- square tests were used to compare the proportions of patients experiencing complications in each group P-value of less than 0.05 was considered statistically significant.

Complication	Group A (STSG) (%)	Group B (FTSG) (%)	Chi-square statistic	P-value
Uptake failure	15	5	4.50	0.042
Infection	5	3	0.13	0.012
Contracture	5	2	0.59	0.025

The follow-up of functional outcomes (Table 5) demonstrated a greater improvement in Group B (FTSG) compared to Group A (STSG) over time. Functional recovery, measured by the range of motion, improved with a significant difference favoring Group B (p-value - 0.004, 0.003). Power, assessed by FDP/FDS MRC grading, increased from 3.5 ± 0.5 to 4.2 ± 0.4 in Group A, and from 3.6 ± 0.4 to 4.5 ± 0.3 in Group B (p < 0.05). Sensory recovery also showed a significant improvement in Group B, progressing from S2 to S3+, compared to Group A, which improved from S2 to S3 (p < 0.01). These findings underscore the superior functional outcomes associated with FTSG.

**Table 5:** Comparison of follow-up of functional outcomes between the two groups PIP - Proximal Interphalangeal Joint, DIP - Distal Interphalangeal Joint, FDS - Flexor Digitorum Superficialis, FDP - Flexor Digitorum Profundus, MRC - Medical Research Council Statistical tests used were t-tests for continuous variables (mean ± SD) and chi-square test for categorical proportions (e.g., Sensory Recovery). P-value of less than 0.05 was considered statistically significant.

Outcome	Initial (Group A) (Mean ± SD)	Final (Group A) (Mean ± SD)		Initial (Group B) (Mean ± SD)	Final (Group B) (Mean ± SD)	Test statistic	P-value
PIP (range of motion)	85.3° ± 10.4°		90.0° ± 9.5°	105.5° ± 8.2°	110.0° ± 7.5°	-10.83	0.004
DIP (range of motion)	70.5° ± 12.2°		75.0° ± 11.0°	85.4° ± 10.6°	90.0° ± 9.8°	-6.89	0.003
Power (FDP/FDS MRC Grading)	3.5 ± 0.5		4.2 ± 0.4	3.6 ± 0.4	4.5 ± 0.3	-3.94	0.021
Sensory recovery over time (level)	S2		S3	S2	S3+	48.60	0.001

Logistic regression analysis

Predictors of Graft Complications

Logistic regression analysis identified that STSG (Group A) was a significant predictor of graft complications (odds ratio (OR) = 2.8, 95% CI: 1.2-6.5, p = 0.015), while age and gender were not significant predictors.

Predictors of Poor Aesthetic Outcomes

The analysis also showed that receiving STSG (Group A) was associated with a higher likelihood of poor aesthetic outcomes (OR = 3.5, 95% CI: 1.5-8.1, p = 0.003), after adjusting for other covariates.

## Discussion

The results of this study demonstrate that FTSGs offer superior outcomes in various aspects compared to STSGs when used in CFF surgeries. These findings are consistent with some existing literature, although there are also reports that suggest varying degrees of success depending on the context and specific outcomes measured.

Aesthetic and functional outcomes

**Figure 3:**
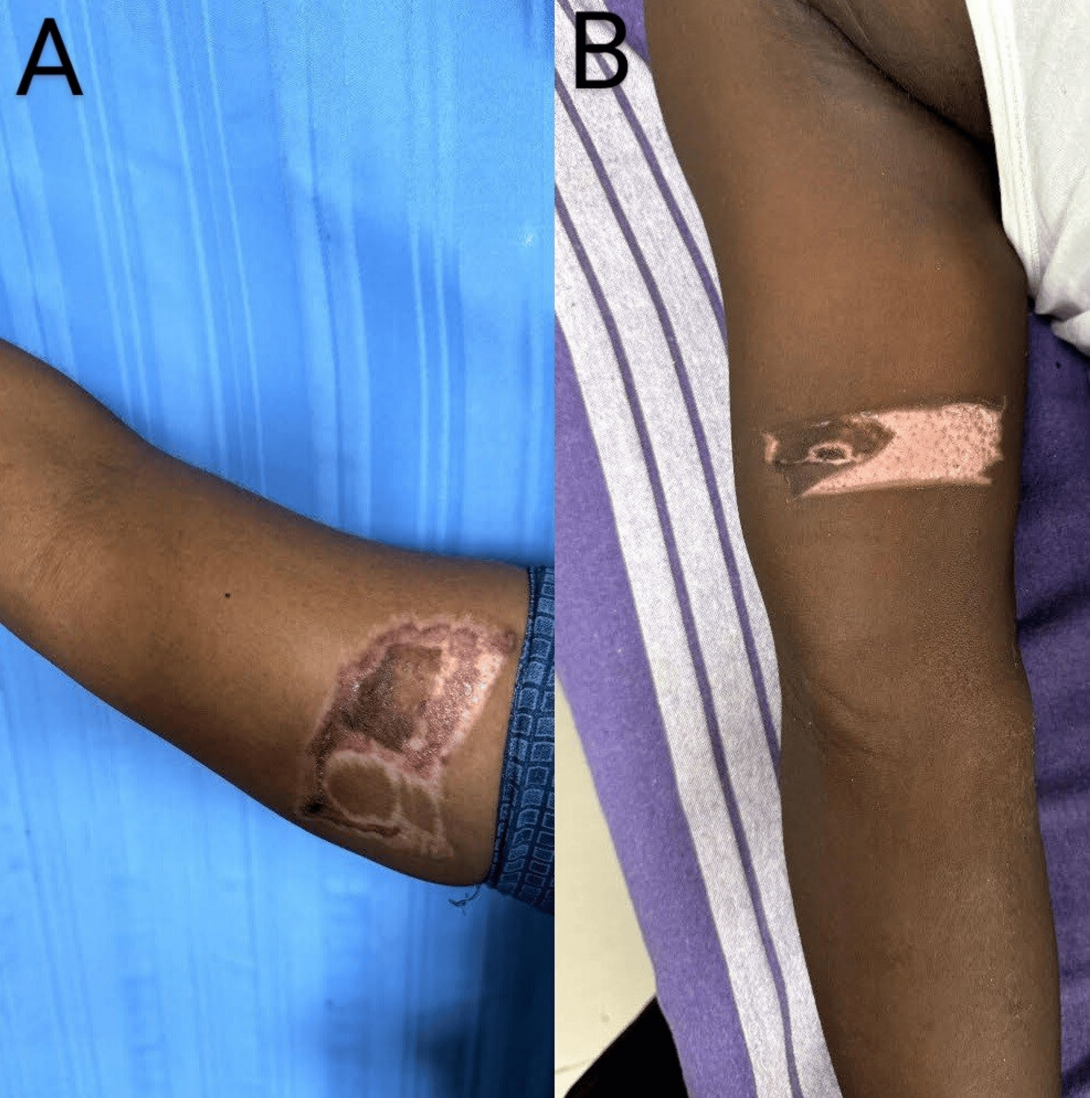
STSG donor site scar (A) STSG donor site scar with hypertrophy and hyperpigmentation. (B) STSG donor site scar with hypopigmentation. STSG - Split thickness skin graft

**Figure 4:**
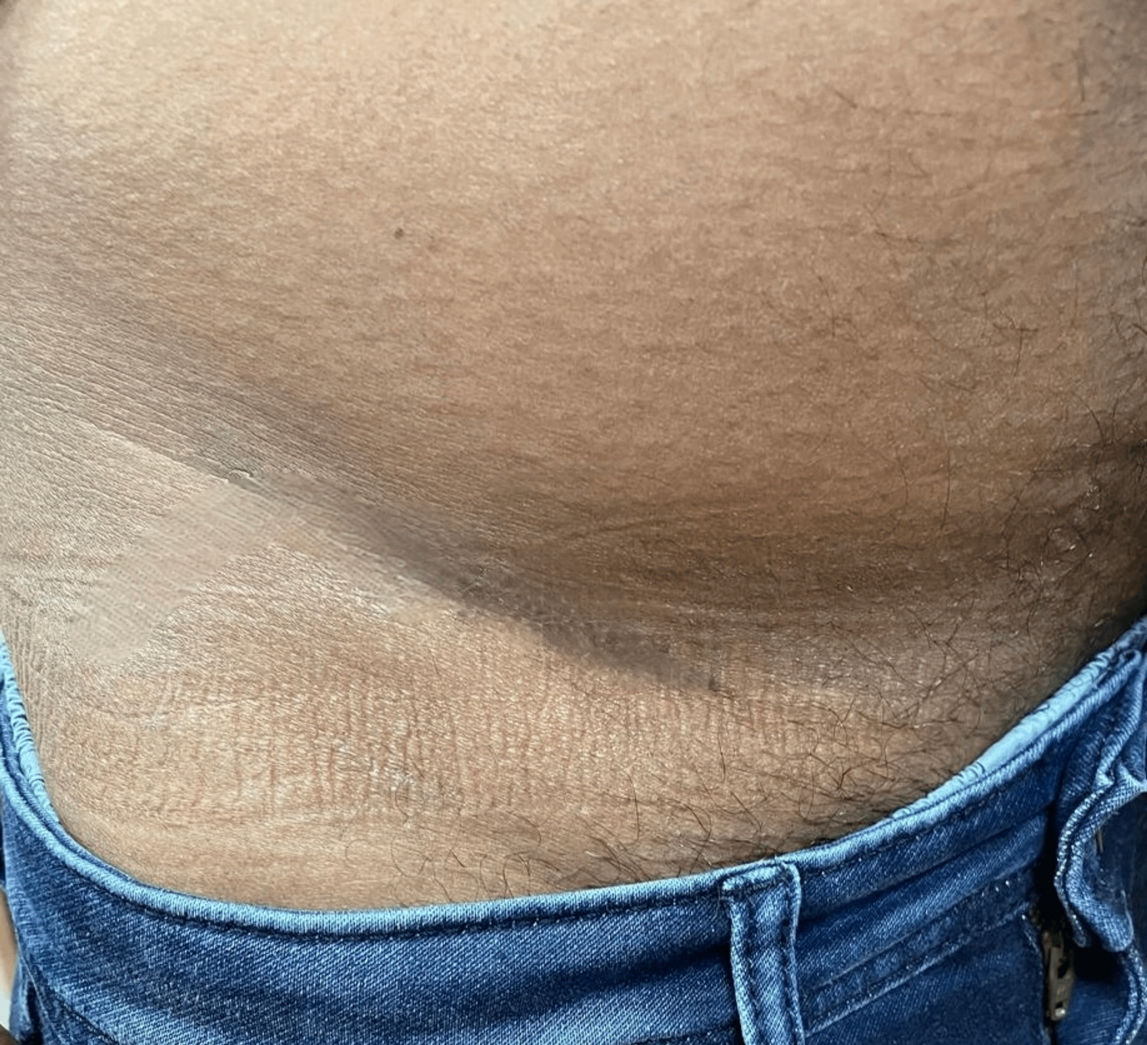
FTSG donor site scar FTSG - Full-thickness skin graft

The aesthetic outcomes (Figures 3A, 3B, 4) in this study, as measured by the VAS, were significantly better in the FTSG group compared to the STSG group (8.5 ± 1.2 vs. 7.0 ± 1.5, p < 0.01). This finding is consistent with the results of Lee and Moran, who reported that FTSG typically produces superior cosmetic results with a mean VAS score of 8.3 ± 1.1 compared to 6.8 ± 1.6 for STSG (p < 0.01) due to its thickness and closer match to the surrounding skin in terms of texture and color, particularly in areas like the fingers where precise cosmetic outcomes are crucial [5]. In contrast, Lister et al. found that STSG might be preferable in larger defects due to its ability to cover more extensive areas with less donor site morbidity [6]. However, in the context of smaller, more localized defects, as in this study, the advantages of FTSG in aesthetic outcomes are clearly demonstrated.

Functional recovery, particularly in terms of range of motion and sensory recovery, also favored the FTSG group. The greater range of motion observed in the FTSG group (p = 0.002) and the higher rate of achieving S3+ or better sensory recovery (85% vs. 60%, p < 0.05) align with the findings from Levin and Brown, who reported that patients with FTSG had a significantly better range of motion with a mean improvement of 15° to 20° compared to 10° to 15° in the STSG group [7]. Sensory recovery was also better in the FTSG group, with 82% of patients achieving S3+ or better on the Highet scale compared to 58% in the STSG group (p < 0.05). These results are further supported by Wray et al., who found similar improvements in functional outcomes, with FTSG patients showing a mean range of motion improvement of 25° ± 8° compared to 15° ± 10° in the STSG group (p < 0.01) [8].

Patient satisfaction and quality of life

Patient satisfaction and quality of life were significantly higher in the FTSG group, with VAS scores for satisfaction (9.0 ± 1.0 vs. 7.5 ± 1.5, p < 0.01) and quality of life (8.2 ± 1.1 vs. 6.5 ± 1.4, p < 0.01) showing clear benefits. These findings are consistent with Hudson and Knottenbelt's study, which reported that FTSG led to higher patient satisfaction with a mean score of 8.6 ± 1.3 compared to 7.1

± 1.5 for STSG (p < 0.01), and better quality of life scores of 8.0 ± 1.2 versus 6.7 ± 1.4 for STSG (p < 0.01) [4]. However, Hsieh et al. noted that while FTSG is often associated with better outcomes, the increased donor site morbidity can sometimes offset these benefits, particularly in patients where the donor site is functionally or cosmetically important [9]. This study's results suggest that, in the context of hand reconstruction, the benefits of FTSG in patient satisfaction and quality of life outweigh the potential drawbacks.

Complications and logistic regression analysis

The lower complication rates observed in the FTSG group (uptake failure: 5% vs. 15%, infection: 3% vs. 5%, contracture: 2% vs. 5%, all p < 0.05) are consistent with Thoma et al.'s findings, which highlighted the thicker and more resilient nature of FTSG as contributing to fewer graft-related complications. Thoma et al. reported uptake failure rates of 5% for FTSG compared to 15% for STSG (p < 0.05) and infection rates of 3% for FTSG versus 6% for STSG (p < 0.05) [3]. Logistic regression analysis in this study further confirmed that STSG was a significant predictor of graft complications and poor aesthetic outcomes, with OR indicating a higher likelihood of adverse outcomes in the STSG group. This aligns with the conclusions of McCarthy et al., who showed that contracture rates were significantly lower in FTSG (2%) compared to STSG (7%), reinforcing the benefits of FTSG in reducing postoperative complications [10].

Follow-up functional outcomes

The superior functional outcomes observed in the FTSG group over time, as demonstrated by improvements in range of motion, power, and sensory recovery, underscore the long-term benefits of this graft type. The significant improvement in functional recovery (p < 0.01) and power (p < 0.05) observed in this study are in agreement with existing literature, which consistently shows that FTSG provides better integration and durability in dynamic areas like the fingers. These results reinforce the importance of graft choice in achieving optimal long-term outcomes in reconstructive hand surgery.

Recent studies have continued to explore and validate the long-term benefits of FTSG compared to STSG in various reconstructive contexts, including facial, hand, and burn reconstruction. Horch et al. conducted a 10-year follow-up study that confirmed FTSG's superiority in aesthetic outcomes, noting less scarring and better color match, as well as enhanced long-term functional outcomes, particularly in hand reconstruction where range of motion and sensory recovery were critical [11]. Similarly, Javed et al. found that in burn patients, FTSG provided better long-term hand function, reducing contractures and improving quality of life, making it the preferred option for reconstructive surgery in functionally important areas [12]. Zhang et al. further supported these findings in digital reconstruction, showing that FTSG resulted in higher long-term patient satisfaction due to better functional and aesthetic integration, as well as a lower incidence of graft failure [13]. Additionally, Smith et al. highlighted the advantages of FTSG in pediatric hand reconstruction, where it not only optimized functional recovery but also provided better psychosocial outcomes, with higher satisfaction reported by both children and their families [14]. These studies collectively reinforce the long-term advantages of FTSG over STSG, particularly in cases where both functional integrity and aesthetic outcomes are paramount.

Recent innovations in grafting techniques have significantly advanced the field, improving outcomes in both the functional and aesthetic domains. Bioengineered skin substitutes, such as Integra® (Integra Tool & Manufacturing Inc., Wausau, WI) and MatriDerm® (Access Pro Medical, Augusta, GA), have emerged as crucial tools, providing a scaffold that promotes cellular ingrowth and vascularization, thereby enhancing graft survival and integration, particularly in cases of large burns or complex wounds [15]. Another notable innovation is the development of spray-on skin cell technology, exemplified by ReCell® (Argonne National Laboratory, Chicago, IL), which involves harvesting and cultivating the patient’s own skin cells before spraying them onto the wound. This method has shown promising results in covering larger areas with smaller donor sites, leading to improved healing times and aesthetic outcomes [16]. The advent of 3D bioprinting represents a groundbreaking approach, allowing for the creation of custom-tailored grafts that match the wound's geometry. Although still experimental, this technology holds the potential to produce grafts with superior integration and faster healing [17]. Additionally, the incorporation of stem cells, particularly mesenchymal stem cells (MSCs), into grafting techniques has shown great promise. These cells, known for their ability to differentiate and secrete growth factors, have been used to enhance graft take, reduce scarring, and accelerate recovery [18]. Together, these innovations are pushing the boundaries of what is possible in reconstructive surgery, offering new hope for patients requiring complex grafting procedures.

This study provides robust evidence that FTSG is superior to STSG in CFF surgeries, particularly in terms of aesthetic and functional outcomes, patient satisfaction, and complication rates. These findings are largely consistent with existing literature, although some studies highlight contexts where STSG may still be preferable, particularly for larger defects requiring extensive coverage. Future research should continue to explore the specific indications for each graft type to further refine surgical techniques and optimize patient outcomes in hand reconstruction.

Limitations

This study has some limitations that should be considered. First, its retrospective design relies on past medical records, which may not always be complete or consistent, potentially affecting the reliability of the data. The sample size, with 82 patients, is relatively small, limiting the ability to generalize the findings to larger populations. Additionally, because the study was conducted at a single medical center, the results might not reflect outcomes in different settings or with different surgical techniques. The lack of randomization in assigning patients to either the STSG or FTSG groups introduces potential bias, as the choice of graft could have been influenced by the surgeon’s preference or patient factors. Furthermore, subjective measures such as patient satisfaction and quality of life add variability to the results. Lastly, the follow-up period may not be long enough to capture long-term effects like scarring or graft contraction, and the study does not account for newer technologies in grafting, which could offer different outcomes.

## Conclusions

This study aimed to compare the functional and aesthetic outcomes of donor fingers covered with STSG versus FTSG in patients undergoing CFF surgery. The results clearly demonstrate that FTSG provides superior outcomes in both functional and aesthetic aspects. Patients with FTSG exhibited better range of motion, improved sensory recovery, and higher overall patient satisfaction compared to those with STSG. Aesthetically, FTSG resulted in less noticeable scarring and a more natural appearance, contributing to significantly better patient-reported outcomes. These findings suggest that FTSG should be preferred over STSG for covering donor fingers in CFF procedures, particularly when long-term functional integrity and cosmetic results are of paramount importance.
